# Refractory pharyngeal ulceration due to cytomegalovirus in a patient with HIV infection: a case report and literature review

**DOI:** 10.1186/s12879-021-05943-w

**Published:** 2021-03-10

**Authors:** Morichika Osa, Akihiro Sato, Maki Sakagami, Masaki Machida, Takao Sato, Ayaka Tsukimori, Shinji Fukushima, Itaru Nakamura, Ryo Akai, Kiyoaki Tsukahara, Hidehiro Watanabe

**Affiliations:** 1 Department of Infectious Disease, Tokyo Medical University Hospital, 6-7-1 Nishi-Shinjuku, Shinjuku-ku, Tokyo, 160-0023 Japan; 2 Division of Infectious Diseases and Respiratory Medicine, Department of Internal Medicine, National Defense Medical College Hospital, 3-2 Namiki, Tokorozawa, Saitama, 359-8513 Japan; 3 Department of Otorhinolaryngology-Head and Neck Surgery, Tokyo Medical University Hospital, 6-7-1 Nishi-Shinjuku, Shinjuku-ku, Tokyo, 160-0023 Japan

**Keywords:** Cytomegalovirus, Pharyngeal ulceration, HIV, Ganciclovir, Case report

## Abstract

**Background:**

Cytomegalovirus (CMV) is an important pathogen among immunocompromised hosts. Typically, CMV in human immunodeficiency virus (HIV) infection causes diseases of the retina, digestive tract, lungs and liver, but there are few cases of CMV infection of the pharynx and larynx.

**Case presentation:**

A 57-year-old man with HIV infection was admitted because of pharyngeal pain. Before and after admission, pharyngeal biopsies guided by laryngeal endoscopy were performed four times, but pathological examination showed nonspecific inflammation, and the cause of pharyngeal ulceration was unclear. Additionally, the ulceration deteriorated after initiation of retroviral therapy. Laryngomicrosurgery was conducted under general anesthesia to remove tissue, and pathological diagnosis confirmed CMV infection. Pathological features included enlargement of the cytoplasm and nucleus in infected cells, and intranuclear bodies called owl’s eye inclusions. Ganciclovir dramatically improved the symptoms and laryngoscopic findings.

**Conclusions:**

This case was diagnosed as pharyngitis and pharyngeal ulceration caused by CMV infection, related to immune reconstitution inflammatory syndrome. In previous reports of CMV-induced pharyngeal or laryngeal ulceration in HIV infection, we found six cases similar to our present case. All cases were diagnosed by biopsy. The present case indicates the importance of biopsy for definitive diagnosis. CMV infection should be considered as a differential diagnosis of pharyngeal ulceration in patients with HIV infection.

## Background

Cytomegalovirus (CMV) is a member of the Herpesviridae that is mainly present in the salivary glands and is not pathogenic in immunocompetent people. However, CMV causes infection in immunocompromised hosts, such as organ transplantation recipients, human immunodeficiency virus (HIV)-infected patients, and people treated with immunosuppressive agents (e.g., chemotherapy, steroids). In particular, CMV in patients with HIV infection causes diseases of the retina, digestive tract, lungs and liver [1]. CMV colitis likely causes ulceration as well as inflammation. However, there are few case reports of CMV infection causing ulceration in the pharynx and larynx. We report a case of pharyngeal ulceration caused by CMV in a patient with HIV infection that required five biopsies for diagnosis.

## Case presentation

A 57-year-old Japanese man visited a nearby hospital because of pharyngeal pain. He was diagnosed with *Candida* pharyngitis and treated with oral amphotericin B. However, because his symptoms did not improve, he was referred to our hospital and scrutinized.

**Fig. 1:**
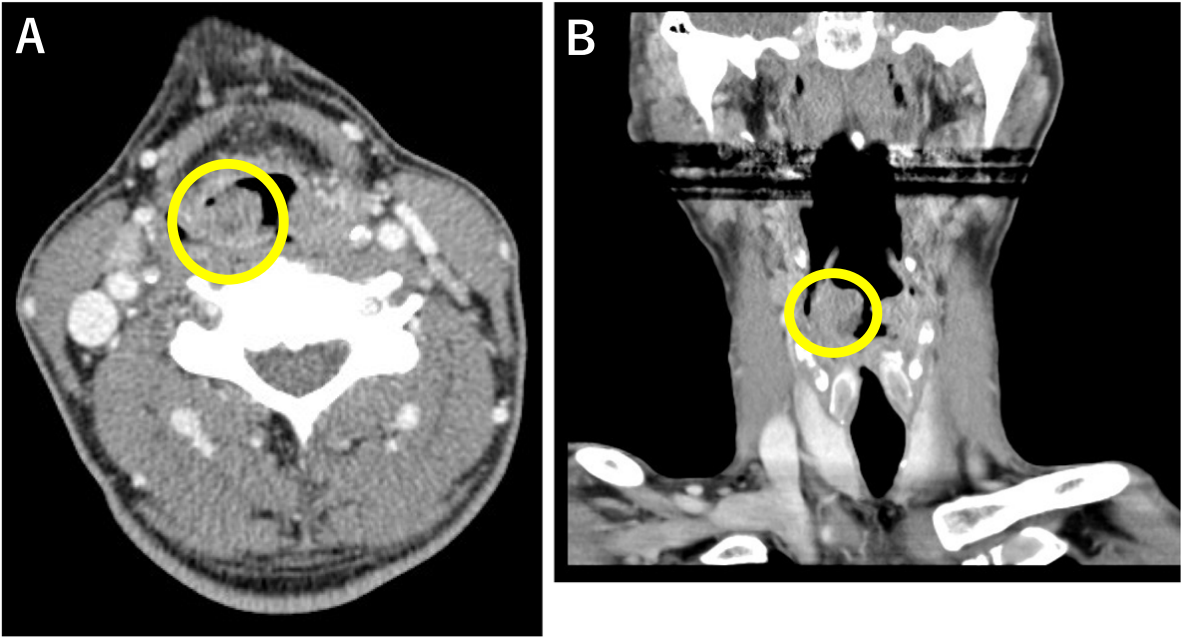
**a**: Axial section of head and neck computed tomography. **b**: Coronal section of head and neck computed tomography. Both images show the right arytenoid edema (yellow circle)

**Fig. 2:**
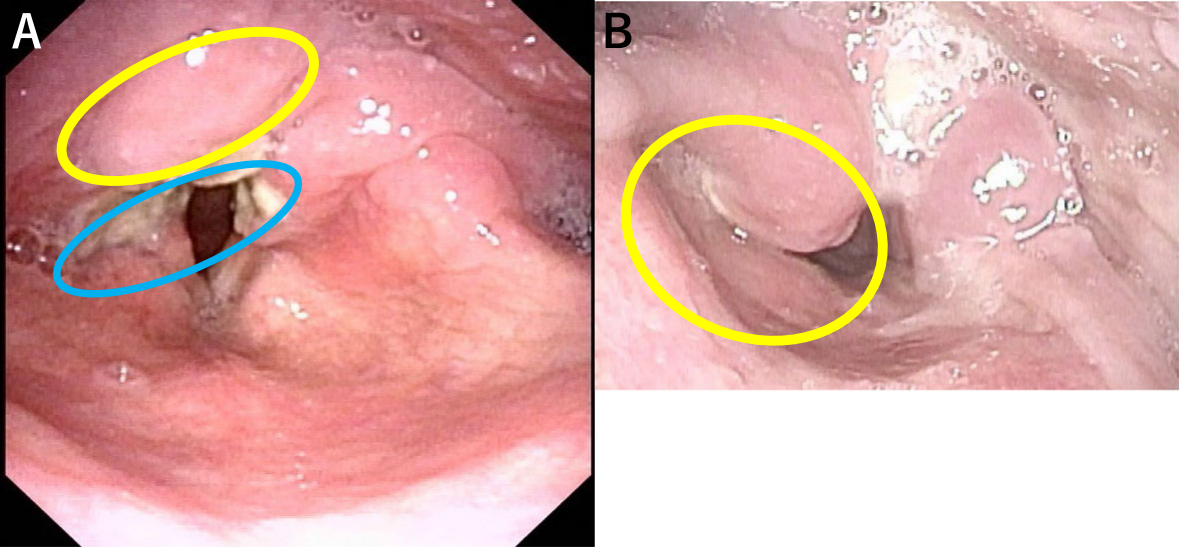
**a**: Initial laryngoscopy findings: right arytenoid edema (yellow circle) and pharyngeal ulceration (blue circle). **b**: Laryngoscopy at admission: right arytenoid edema had deteriorated

Two months after symptom onset, the patient was afebrile, and physical examination revealed redness of the pharynx. HIV rapid antigen test and polymerase chain reaction were positive, and rapid plasma reagin test (28.8 R.U) and *Treponema pallidum* latex-agglutination test (4476.0 T.U) were also positive. We suspected *Candida* pharyngitis or *Treponema pallidum* infection. Amoxicillin and fluconazole had been administered for 4 weeks and 2 weeks, respectively, but pharyngeal pain deteriorated. Additionally, at the time of presentation, biopsy was performed by an otolaryngologist and there was no evidence of secondary syphilis from the biopsy specimen. Three months after symptom onset, he was admitted to our hospital. On physical examination, the patient was afebrile with a temperature of 36.7 °C, normal blood pressure of 113/78 mmHg, normal heart rate of 88 beats/min, and normal SpO_2_ of 97% (room air). HIV-1 viral load and CD4 counts were 3.1*10^5 cps/mL and 11 cells/μL, respectively. Results of other laboratory and imaging studies are shown in Table 1. After admission, the symptoms were resistant to treatment and hoarseness gradually developed. Right arytenoid edema was shown by computed tomography (Fig. 1a, b) and nasopharyngolaryngoscopy (Fig. 2a, b). At this time, blood CMV pp65 antigen was negative. To investigate CMV infection, polymerase chain reaction (PCR) is the standard for diagnosis. In our case, CMV PCR was not performed because this test was not covered by national health insurance in Japan; therefore, we tested for blood CMV antigen, which is comparable to CMV PCR [2].

**Table 1:** Laboratory data on admission

Peripheral blood			Blood chemistry			Serology		
WBC	7100	/μL	T-Bil	0.14	mg/dL	CRP	3.88	mg/dL
Hb	10.2	g/dL	AST	50	U/L	sIL-2R	691	U/mL
PLT	22.9 × 10^4^	/μL	ALT	60	U/L	RPR	26.5	R.U
			LDH	254	U/L	TPLA	4187	T.U
			γ-GTP	74	U/L	IGRA	(−)	
			BUN	33.5	mg/dL	HBs-Ag	(−)	
			Cr	1.16	mg/dL	HCV-Ab	(−)	
						Toxo-IgG	(−)	

*Abbreviations*: *ALT* alanine aminotransferase, *AST* aspartate aminotransferase, *BUN* blood urea nitrogen, *Cr* creatinine, *CRP* C-reactive protein, *Hb* hemoglobin, *HBs-Ag* hepatitis B surface antigen, *HCV-Ab* hepatitis C antibody, *IGRA* interferon-γ release assay, *LDH* lactate dehydrogenase, *PLT* platelets, *RPR* rapid plasma reagin test, *R.U*. RPR units, *sIL-2R* soluble interleukin-2 receptor, *T-Bil* total bilirubin, *Toxo-IgG Toxoplasma* IgG, *TPLA Treponema pallidum* latex-agglutination, *T.U*. titer units, *WBC* white blood cells

An otolaryngologist recommended betamethasone for treatment of laryngeal edema but ulceration and laryngeal edema worsened, and tracheostomy was performed. Blood tests, computed tomography and consultation with an ophthalmologist showed that the patient was negative for active meningeal tuberculosis, cryptococcal meningitis and other opportunistic infections, including CMV infection such as retinitis, gastroenteritis, pneumonitis and hepatitis. Therefore, we started antiretroviral therapy (ART) with abacavir (600 mg), lamivudine (300 mg) and dolutegravir (50 mg), according to the Japanese national protocol. To evaluate the pharyngeal ulceration and edema, biopsies were performed four times after admission. However, the cause of pharyngeal ulceration remained unclear and the symptoms did not improve at all. Three weeks after administration of ART, CD4 cell count increased to 53/μL and HIV-1 viral load decreased to 5.9*10^2cps/mL.

**Fig. 3:**
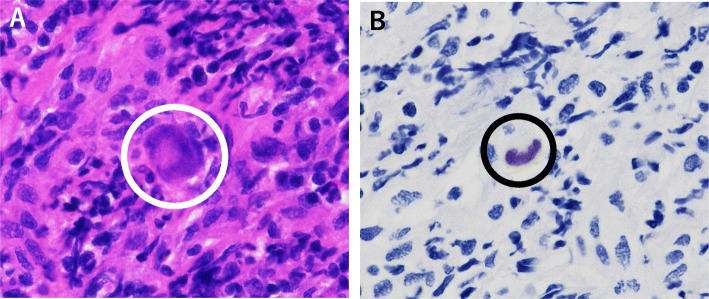
**a**: Hematoxylin–eosin: histopathological features of lesion demonstrating enlargement of the cell nucleus (white circle). **b**: Positive immunostaining for CMV by monoclonal antibody (black circle)

Additionally, blood CMV pp65 antigen was positive, although it was negative upon admission. CMV can infect the retina, digestive tract, lungs and liver. To investigate these targets of CMV infection, the patient consulted an ophthalmologist and gastroenterologist, and underwent chest X-ray and liver blood tests, respectively. Colonoscopy revealed CMV enteritis, and oral valganciclovir was started. At the same time, tissue biopsy by laryngomicrosurgery was performed under general anesthesia, and we finally found that pharyngeal ulceration was caused by CMV infection (Fig. 3a, b). Therefore, oral valganciclovir was switched to ganciclovir injection. After treatment with ART and ganciclovir, the symptoms dramatically improved. This case was diagnosed as pharyngitis and pharyngeal ulceration caused by CMV infection. Additionally, this case could have been caused by immune reconstitution inflammatory syndrome (IRIS). Because CMV PCR was not performed during the clinical course, it is difficult to confirm whether CMV infection was related to IRIS or newly occurring. One hypothesis is that latent CMV infection caused IRIS after administration of ART.

## Discussion and conclusions

We present the case of an HIV-infected man with CMV pharyngeal ulceration that may have been caused by IRIS. Even though several biopsies were performed, the cause of pharyngeal ulceration was not detected. Eventually, after initiation of ART, CMV pharyngeal ulceration was diagnosed by biopsy under general anesthesia. There were three reasons why the diagnosis was difficult. First, CMV infection was not suspected because C7HRP was negative at the time of admission. Second, the patient had no typical manifestations of CMV infection, such as retinitis, colitis or hepatitis. Third, the amount of collected tissue might have been too small for diagnosis. Also, C7HRP became positive after ART. Therefore, this case was thought to have been caused by IRIS.

Typically, CMV causes infection in the retina, digestive tract, lungs and liver [1]. However, pharyngeal and laryngeal ulceration is not typical. Moreover, the present case seems to be rare because it showed CMV pharyngeal ulceration caused by IRIS without CMV retinitis, pneumonitis and hepatitis.

To identify previous cases of CMV pharyngeal or laryngeal ulceration, we searched PubMed using the keywords “Cytomegalovirus”, “CMV”, “HIV”, “Pharyngeal” and “Laryngeal”. Additionally, we searched the references of published reports. Seven cases were identified, and they are listed, along with the present case, in Table 2. Two studies were excluded because they did not include enough information for evaluation [6, 7]. The average age of the patients was 40.3 ± 9.6 years, six were male, and one had died. CD4 cell count, HIV-1 RNA viral load and CMV DNA or antigen test was available in two cases. In one case, CD4 cell count was 2/ul, HIV-1 RNA viral load was 5.5*10^5/mL and CMV antigen was positive. In our case, CD4 cell count was 11/uL, HIV-1 RNA viral load was 3.1*10^5/mL and CMV antigen was positive. Two cases were not treated and regressed spontaneously, but the others were treated by ganciclovir or valganciclovir. In terms of treatment period, two cases and our case were treated for 2 weeks, one case was treated for 8 weeks. Diagnosis was achieved with one biopsy in five cases and two biopsies in one case. Additionally, it is only our case that describes CMV pharyngeal and laryngeal ulceration caused by IRIS, without typical manifestations such as retinitis, pneumonitis and hepatitis.

**Table 2:** Summary of CMV pharyngeal or laryngeal ulceration with HIV infection in the current and previous cases

No. (Ref)	Age, yr	Sex	CD4 cell count (/μL)	HIV RNA (copies/mL)	CMV DNA or CMV antigen	Site	No. of biopsies	Treatment	Treatment period	Outcome	IRIS
1 [3]	27	M	NA	NA	NA	P	1	GCV	2 weeks	Improved	NA
2 [3]	43	M	NA	NA	NA	L	2	GCV	2 weeks	Improved	NA
3 [4]	36	M	NA	NA	NA	O,P	1	None	NA	Improved	NA
4 [4]	42	M	NA	NA	NA	C,D,E,P	1	GCV	8 weeks	Death	NA
5 [4]	45	M	NA	NA	NA	D,L,O,P	1	GCV	NA	Improved	NA
6 [5]	33	F	2	5.5*10^5	Antigen positive	P	1	None	NA	Improved	NA
Present case	57	M	11	3.1*10^5	Antigen positive	D,P	5	GCV,VGCV	2 weeks	Improved	+

*C* central nervous system, *CMV* cytomegalovirus, *D* digestive tract, *E* eye, *F* female, *GCV* ganciclovir, *HIV* human immunodeficiency virus, *IRIS* immune reconstitution inflammatory syndrome, *L* larynx, *M* male, *NA* not available, *O* oral cavity, *P* pharynx, *VGCV* valganciclovir

Our case required five biopsies for diagnosis. In one of the previous reports, the authors stated that histological diagnosis is vital for diagnosis of oropharyngeal ulcers caused by CMV because these lesions show various microscopic findings [3]. There are many differential diagnoses of pharyngeal ulceration and it is difficult to diagnose. If patients with HIV infection show refractory pharyngeal or laryngeal pain and ulceration, biopsy should be performed. Additionally, this case suggests that CMV IRIS could be the differential diagnosis when refractory pharyngeal or laryngeal ulceration is seen. In conclusion, if a patient has oropharyngeal ulcer with HIV infection, CMV infection should be considered as differential diagnosis.

## Data Availability

The datasets used and/or analyzed during the current study are available from the corresponding author on reasonable request.

## Abbreviations

CMV: Cytomegalovirus
HIV: Human immunodeficiency virus
IRIS: Immune reconstitution inflammatory syndrome
PCR: Polymerase chain reaction
RPR: Rapid plasma reagin test
